# Safety, Tolerability, and Immunogenicity of aH5N1 Vaccine in Adults with and without Underlying Medical Conditions

**DOI:** 10.3390/vaccines12050481

**Published:** 2024-04-30

**Authors:** Tomas Jelinek, Tino F. Schwarz, Emil Reisinger, Peter Malfertheiner, Eve Versage, Esther Van Twuijver, Matthew Hohenboken

**Affiliations:** 1 Berlin Center for Travel and Tropical Medicine, 10117 Berlin, Germany; 2 Department of Laboratory Medicine, Klinikum Würzburg Mitte, Standort Juliusspital, 97070 Würzburg, Germany; tino.schwarz@kwm-klinikum.de; 3 Medical Faculty, Universitätsmedizin Rostock, 18057 Rostock, Germany; emil.reisinger@uni-rostock.de; 4 Department of Gastroenterology, Hepatology and Infectious Diseases, Otto von Guericke University, 39106 Magdeburg, Germany; peter.malfertheiner@med.ovgu.de; 5 Seqirus, Clinical Development, Cambridge, MA 02139, USA; eve.versage@seqirus.com; 6 Seqirus, Clinical Development, 1105 BJ Amsterdam, The Netherlands

**Keywords:** pandemic influenza, pandemic influenza vaccine, adjuvanted H5N1 vaccine, high-risk medical conditions, older adults

## Abstract

Influenza pandemics pose a serious risk to the global population, with the potential for high morbidity and mortality. An adjuvanted H5N1 vaccine (aH5N1) has been approved for prophylaxis against the avian influenza virus H5N1, which is a likely cause of future pandemics. In this phase-III, stratified, randomized, controlled, observer-blind, multicenter study, we evaluated the safety and immunogenicity of aH5N1 in four separate groups of adults: adults 18–60 years of age who were healthy or had high-risk medical conditions and older adults ≥61 years of age who were healthy or had high-risk medical conditions. Subjects were randomly assigned to aH5N1 or the comparator, adjuvanted trivalent seasonal influenza vaccine (aTIV). Antibody responses to aH5N1 were increased in all four subgroups and, within each age stratum, largely consistent between healthy subjects and those with medical conditions. Injection-site pain was reported by 66–73% of younger and 36–42% of older–aH5N1 recipients, and fatigue and myalgia were reported by 22–41% of subjects across age and health subgroups. No serious adverse events or deaths were considered related to the study vaccine. In conclusion, aH5N1 increased antibody responses regardless of age or health status and demonstrated a clinically acceptable safety and tolerability profile.

## 1. Introduction

Pandemic influenza outbreaks spread quickly and cause substantial global morbidity and mortality [1,2,3]. Older people and those with chronic respiratory or cardiovascular diseases are often most vulnerable to seasonal influenza complications, but in a pandemic, younger, healthier people are also at risk [3]. The avian-origin H5N1 strain is less infectious to humans than the novel A/H1N1 influenza strain that caused the 2009 “swine flu” pandemic, but the mortality rate in humans infected with H5N1 is high. Of 878 H5N1 cases reported to the World Health Organization (WHO) between 2003 and 2023, 52% resulted in death [4].

As the primary means of reducing influenza pandemic-associated morbidity and mortality, vaccines play a central role in the global response to pandemics [5,6]. Several health authorities have established goals to begin manufacture of pandemic strain-specific vaccines within 100 days after the appearance of a pandemic threat and to vaccinate as many people as possible within 6 months of a pandemic declaration [2,7,8]. However, the development of antigenically matched vaccines is a time-consuming process that may require 4–6 months, even under the best of circumstances. During interpandemic periods, the production of pre-pandemic vaccines based on likely pandemic-causing candidates permits the vaccination of essential workers and vulnerable populations before antigenically matched vaccines become available [5,6,9,10]. Adjuvants such as MF59 (Seqirus Inc., Boston, MA, USA), a squalene-based oil-in-water emulsion, increase the immunogenicity of vaccines and may also have an antigen-sparing effect, which reduces the amount of antigen needed in each vaccine dose [6,11,12]. An egg-based, adjuvanted H5N1 vaccine (aH5N1 Aflunov^®^, Seqirus Inc.) is approved for immunization against the H5N1 influenza A virus in individuals ≥18 years of age for prophylaxis before a pandemic is declared.

To fulfill a post-authorization commitment to collect aH5N1 clinical data in adult (18–60 years of age) and older (≥61 years of age) individuals with comorbid conditions, we sought to evaluate the safety, tolerability, and immunogenicity of two doses of aH5N1 in a study population that was divided according to vulnerability to influenza infection and complications—that is, younger and older subjects and healthy subjects and those with underlying medical conditions that put them at higher risk of influenza complications [13,14]. As an active control, we used a licensed MF59-adjuvanted trivalent influenza vaccine (aTIV; Fluad^®^, Seqirus, Inc.).

## 2. Materials and Methods

### 2.1. Study Design

In this phase-III, stratified, randomized, controlled, observer-blind, multicenter study, two doses of aH5N1 or the comparator, aTIV, were administered 3 weeks apart to adults aged 18–60 years and older adults aged ≥61 years, with a follow-up of 7 months, at six sites in Germany. The study was designed, implemented, and reported in accordance with the International Council for Harmonisation of Technical Requirements for Pharmaceuticals for Human Use (ICH) Harmonised Tripartite Guideline for Good Clinical Practice (GCP), applicable local regulations, the Declaration of Helsinki, and all subjects provided written, informed consent.

### 2.2. Subjects

Eligible subjects were male or female persons ≥18 years of age with a life expectancy of at least 1 year after study entry. Participants with at-risk medical conditions were identified based on clinical evaluation at study entry to determine the presence of the following underlying medical conditions: chronic pulmonary disease, cardiovascular disease, peripheral vascular disease, diabetes, and/or renal impairment. Subjects with these conditions were categorized at enrollment according to the Charlson Comorbidity Index (CCI).

Subjects were excluded if they had cognitive impairment or psychiatric disease that would interfere with study participation, severe neurologic or seizure disorder, or history of Guillain–Barré syndrome, HIV, or previous receipt of an H5N1 vaccine (see Supplementary Materials for the complete list of inclusion and exclusion criteria).

A sample size of 540 was planned based on safety data from previous studies. Randomization was designed so that more people would receive the vaccine of interest (aH5N1), with greater weighting toward subjects with the above-listed medical conditions in order to collect data on the clinical effects of aH5N1 in vulnerable populations and to achieve enrollment of approximately 30 individuals in each aTIV cohort. Thus, a total of 540 subjects with ≥1 prespecified medical condition (as listed above) were randomly assigned to aH5N1 or aTIV in a 5:1 ratio within each age stratum (18–60 or ≥61 years), whereas healthy subjects within each age stratum were randomized 2:1 to aH5N1 or aTIV. Each age stratum consisted of 270 subjects.

### 2.3. Vaccine Administration

Each 0.5 mL dose of the aH5N1 vaccine contained 0.25 mL of the adjuvant MF59 and approximately 7.5 μg HA of A/turkey/Turkey/1/2005(H5N1)-like (NIBRG-23) influenza antigen. Each 0.5 mL dose of aTIV also contained 0.25 mL of MF59; the purified viral envelope glycoprotein neuraminidase (NA); and approximately 15 μg HA each of A/California/7/2009 (H1N1)pdm09-like virus, A/Texas/50/2012 (H3N2)-like virus, and B/Massachusetts/2/2012-like virus from the B/Yamagata lineage, as recommended by the WHO for the 2014–2015 Northern Hemisphere influenza season [15].

Participating subjects received two doses of assigned study vaccine, administered as two intramuscular injections 3 weeks apart (on Days 1 and 22) to the deltoid muscle, preferably of the nondominant arm. Both vaccines were provided in prefilled syringes, each with an injectable volume of approximately 0.5 mL, which were administered by unblinded study staff.

### 2.4. Procedures

Blinded study staff performed all study-related procedures (except vaccine administration), monitoring, and safety assessments. For ≥30 min after each vaccination, all subjects remained under medical supervision at the study site for safety monitoring. Subjects received diary cards for the recording and collection of information on post-vaccination reactions, adverse events (AEs), and medications or other vaccines received during the treatment period (Days 1–43), which included three clinic visits and four calls to remind patients to fill out diary cards. The follow-up period (Days 44–202) included one clinic visit and four safety calls. Blood samples were collected for immunogenicity assessments before vaccination on Days 1 and 22, on Day 43, and on Day 202.

Over the 7 days following each dose (Days 1–7 and 22–28), subjects recorded all solicited and unsolicited AEs and medications and/or other vaccines given within these time periods on diary cards. During Days 8–22 and Days 29–43, only unsolicited AEs, solicited AEs that continued beyond Day 7 or 28, and medications or vaccines given within these intervals were recorded until the next clinic visit (Days 22 and 43, respectively). Study staff documented a subset of unsolicited AEs during the follow-up period (Days 44–202) by interviewing the subject and/or reviewing available medical records.

### 2.5. Endpoints

#### 2.5.1. Immunogenicity

Antibody responses were evaluated using the 2007 Committee for Medicinal Products for Human Use (CHMP) criteria [16,17]. The primary immunogenicity endpoint was the antibody response to the homologous H5N1 strain as measured by hemagglutination inhibition (HI). Overall, three immunogenicity assays were used, consistent with prior clinical studies and the historical serology sensitivity of avian influenza assays.

Secondary endpoints included homologous antibody responses evaluated with geometric mean areas (GMAs) determined by the single radial hemolysis (SRH) assay on Days 22 and 43; geometric mean ratios (GMR) determined from GMAs for Day 22/Day 1 and Day 43/Day 1; and SRH seroconversion and SRH area ≥ 25 mm^2^ on Days 22 and 43. SRH seroconversion was defined as an SRH area ≥ 25 mm^2^ for subjects who were seronegative at baseline (Day 1 SRH area ≤3.997 mm^2^) or a significant (at least 50%) increase in SRH area for subjects who were seropositive at baseline (SRH area >3.997 mm^2^).

Exploratory endpoints included the above-listed SRH assessments on Day 202 and microneutralization (MN)-assay-determined geometric mean titers (GMTs) and GMR values on Days 1, 22, 43, and 202; percentages of subjects with MN titers ≥1:10, ≥1:40 and ≥1:80 on Days 1, 22, 43, and 202; and a ≥four-fold increase in MN titer on Days 22, 43, and 202. In addition, antibody responses to the heterologous A/H5N1 strains A/Vietnam/1203/2004XPR8 and A/Anhui/01/2005XPR8 IBCDC-RG5 were tested using a similar set of endpoints.

Immunogenicity was established if all three of the following CHMP criteria for each respective age group were met: the percentage of subjects achieving HI or SRH seroconversion was >40%, the percentage achieving an HI titer ≥1:40 or SRH area ≥ 25 mm^2^ was >70%, and the HI and SRH Day 43/Day 1 GMR was >2.5 for subjects 18–60 years of age and if the same three measures were >30%, >60%, and >2.0, respectively, for subjects ≥61 years of age. There are no CHMP criteria for MN.

#### 2.5.2. Safety

Safety endpoints included the percentages of subjects with the following: solicited local and systemic AEs and the use of antipyretics or analgesics within 7 days following each vaccination and during specified time intervals after vaccination (30 min, 6 h through 3 days, 4 h through 7 days, and 6 h through 7 days); any unsolicited AEs reported within 21 days after each vaccination; and serious AEs (SAEs), new-onset chronic diseases (NOCDs), medically attended AEs, adverse events of special interest (AESIs), and AEs leading to study withdrawal from the study collected from Day 1 through 202.

Solicited local AEs included injection-site erythema, injection-site induration, injection-site ecchymosis, and injection-site pain. Solicited systemic AEs included loss of appetite, nausea, fatigue, generalized myalgia, generalized arthralgia, headache, shivering/chills, vomiting, diarrhea, and body temperature ≥38.0 °C.

### 2.6. Statistical Methods

A sample size of 540 was planned based on safety data from previous studies. The immunogenicity full analysis set (FAS) included all enrolled subjects who were randomized and received at least one study vaccination and provided immunogenicity data. The per-protocol set (PPS) included all FAS subjects who received the vaccine to which they were randomized at the scheduled time points, who had no major protocol deviations leading to exclusion as defined prior to unblinding/analysis, and who were not excluded prior to unblinding or analysis.

Data were analyzed descriptively; no formal statistical testing was planned or performed. All statistical analyses for HI and MN titers and SRH areas were performed on logarithmically (base 10) transformed HI, SRH, and MN values that were assumed to follow normal distribution and were analyzed using an analysis of covariance (ANCOVA) model that included the vaccine-group effect and the log-transformed prevaccination antibody titer or area as independent variables. Analyses were done by vaccine group, age stratum, and health status. Adjusted GMTs, GMRs, and two-sided 95% confidence intervals (CIs) were calculated univariately and completed by providing minimum, maximum, and median titers for the different analysis groups.

Safety data were summarized for all enrolled subjects.

## 3. Results

### 3.1. Study Population

**Figure 1:**
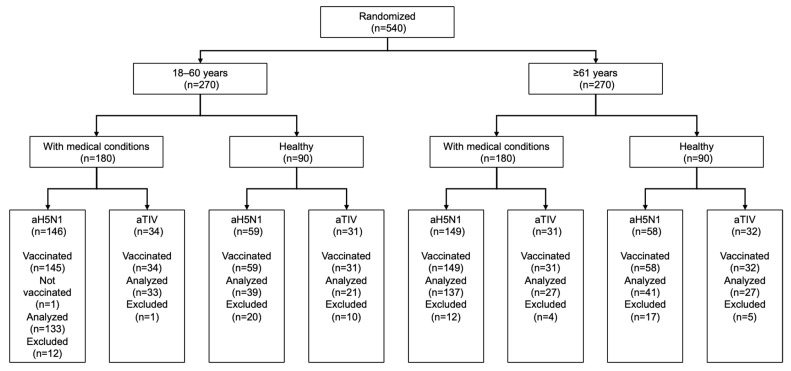
Subject disposition.

Between 10 April 2014 and 15 September 2014, 540 subjects were enrolled and randomly assigned to aH5N1 or aTIV within their respective age and health status groups (Figure 1). A follow-up was completed on 2 April 2015. As shown in Table 1, subjects were predominantly white and not Hispanic. Across age groups, most subjects in the healthy subgroup were female, whereas most participants with underlying medical conditions were male. Diabetes was the most common condition, and subjects with medical conditions had a higher mean body mass index (BMI) than healthy participants.

### 3.2. Immunogenicity

#### 3.2.1. Homologous Strain (A/turkey/Turkey/1/2005)

**Figure 2:**
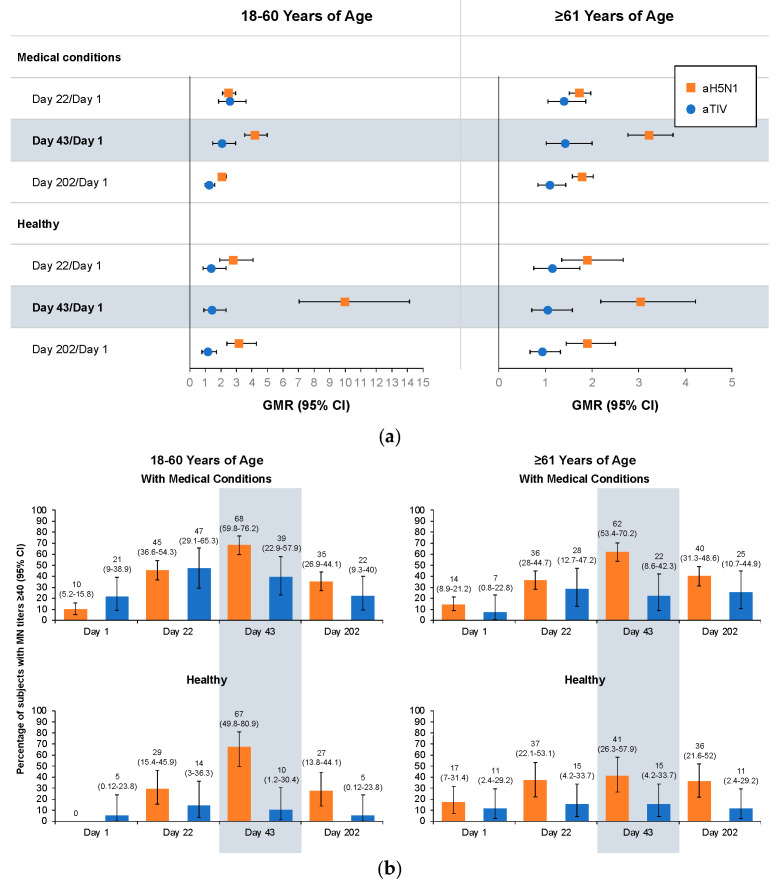

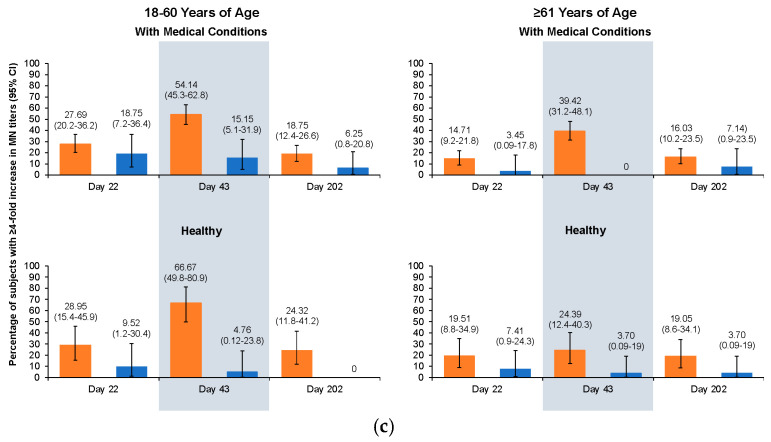
Antibody responses as measured by microneutralization (MN) on Days 1, 22, 43, and 202 in healthy subjects and subjects with medical conditions who were 18–60 or ≥61 years of age at the time of vaccination. aH5N1 = adjuvanted H5N1 vaccine; aTIV = adjuvanted trivalent seasonal influenza vaccine. (**a**) Geometric mean ratios (GMR) of MN titers determined for Day 22/Day 1, Day 43/Day 1, and Day 202/Day 1. (**b**) Percentages of subjects achieving MN titers ≥40 on Days 1, 22, 43, and 202. (**c**) Percentage of subjects with >4-fold increase in MN titer on Days 22, 43, and 202.

The MN and SRH assays used in this study were validated as sensitive and accurate; however, the HI assay could not be validated to the desired level of sensitivity or accuracy at dilutions below 1:40 (HI results are reported in Table S1). Day 43/Day 1 GMRs were greater among aH5N1 than aTIV recipients in all health and age subgroups, and vaccine group differences remained through Day 202 (Figure 2a). Significantly more subjects in the aH5N1 subgroups achieved MN titers ≥40 or had a ≥four-fold increase in MN titers on Day 43 (Figure 2b,c). MN titers ≥10, ≥20, and ≥80 in each subgroup appear in Table S2.

**Figure 3:**
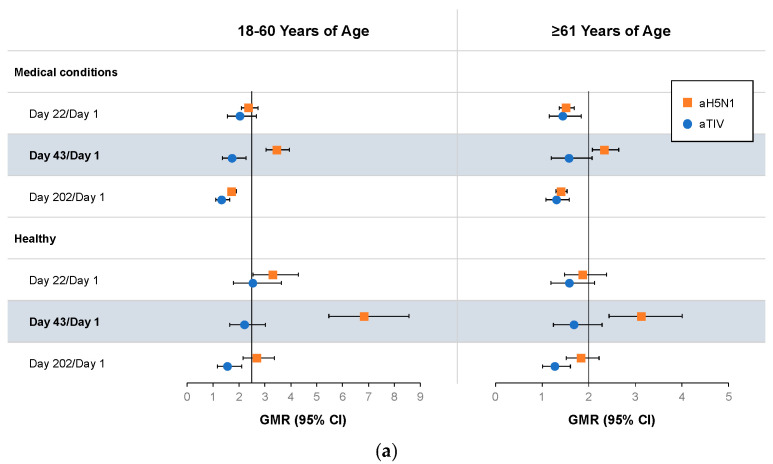

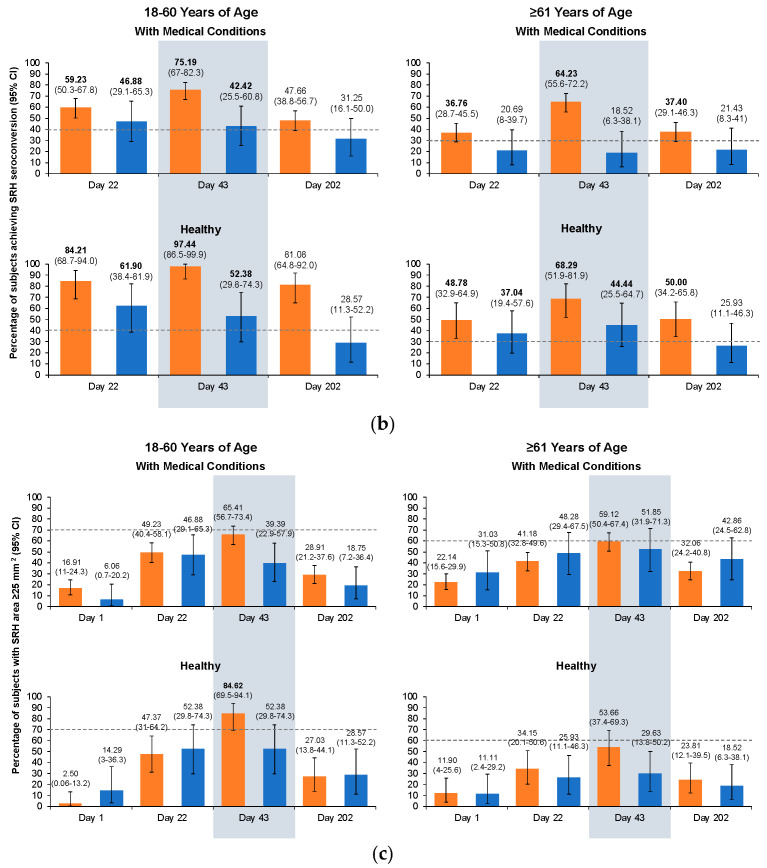
Antibody responses as measured by serial radial hemolysis (SRH) on Days 1, 22, 43, and 202 in healthy subjects and subjects with medical conditions who were 18–60 or ≥61 years of age at the time of vaccination. aH5N1 = adjuvanted H5N1 vaccine; aTIV = adjuvanted trivalent seasonal influenza vaccine. (**a**) Geometric mean ratios (GMR) of geometric mean areas (GMA) determined for Day 22/Day 1, Day 43/Day 1, and Day 202/Day 1. Vertical lines indicate former Committee for Medicinal Products for Human Use (CHMP) criteria for each age group (18–60 years: >2.5; ≥61 years: >2.0). (**b**) Percentages of subjects achieving SRH seroconversion on Days 22, 43, and 202. Dotted lines represent former CHMP criteria for each age group (18–60 years: >40%; ≥61 years: >30%); boldface indicates CHMP criteria were met. (**c**) Percentage of subjects with seroprotection (SRH area ≥ 25 mm^2^) on Days 1, 22, 43, and 202. Dotted lines represent former CHMP criteria for each age group (18–60 years: >70%; ≥61 years: >60%); boldface indicates CHMP criteria were met.

On Day 43, the GMR values determined with the SRH assay were >2.5 among all subjects 18–60 years of age and >2 in subjects ≥61 years of age who received aH5N1, including subjects with underlying medical conditions, as well as healthy subjects (Figure 3a). Seroconversion was achieved by >70% of aH5N1 recipients aged 18–61 years and by >60% of those ≥61 years of age regardless of health status (Figure 3b). An SRH area ≥ 25 mm^2^ was achieved by 84.62% of healthy subjects 18–60 years of age who received aH5N1; <70% of the other subgroups met this criterion (Figure 3c). Healthy subjects in the 18–60 years of age stratum vaccinated with aH5N1 met all three former CHMP criteria for pandemic influenza vaccines as assessed by SRH on Day 43. Subject groups with underlying medical conditions met two of the three former CHMP criteria for pandemic influenza vaccine immunogenicity (seroconversion and GMR), as did the older age stratum of healthy subjects.

#### 3.2.2. Heterologous Strains

aH5N1-induced antibody responses to the heterologous strains Vietnam/2004 and Anhui/2005 were greater in younger than older subjects and in healthy subjects vs. those with medical conditions (Table 2).

### 3.3. Safety

The safety analysis included data from 539 subjects. The percentage of subjects with at least one AE (i.e., solicited or unsolicited AEs reported by >5% of subjects, excluding SAEs) during the 202-day study period was 74.5% in the aH5N1 group and 85.2% in the aTIV group. The percentage of subjects with at least one solicited AE after any vaccination was 75.2% in the aH5N1 group and 85.2% in the aTIV group. In total, 70 SAEs in 48 subjects were reported in 11.0% and 2.3% of subjects in the aH5N1 and aTIV groups, respectively (Table 3). None of the SAEs were considered to be vaccine-related, and most occurred in subjects with medical conditions. Four deaths, none considered vaccine-related, were reported in subjects with medical conditions (two in each age stratum) and were attributed to acute cardiac failure, sudden cardiac arrest, pneumonia and multiple organ failure, and septic shock. All four deaths occurred in the aH5N1 group approximately 4–6 months after the second vaccination.

**Figure 4:**
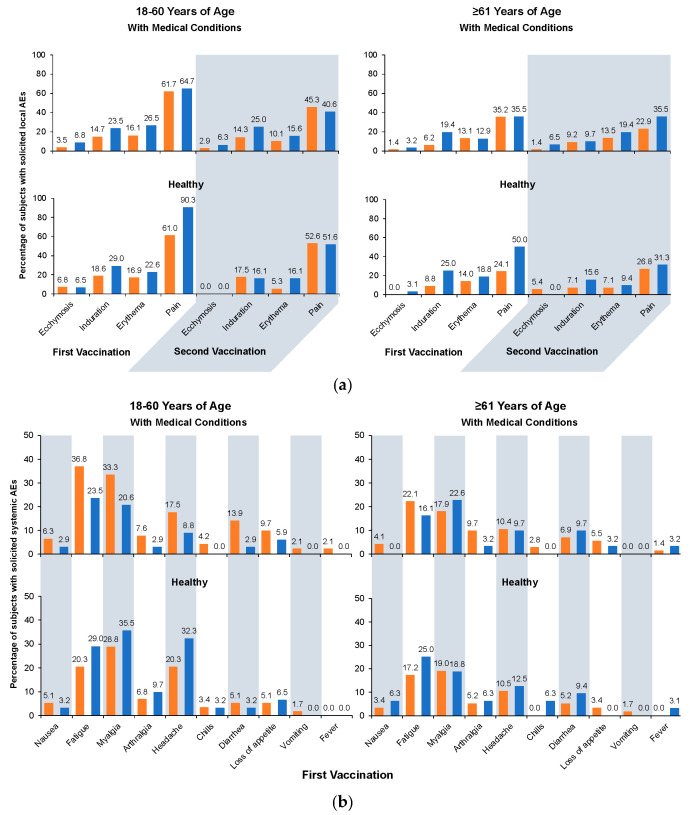

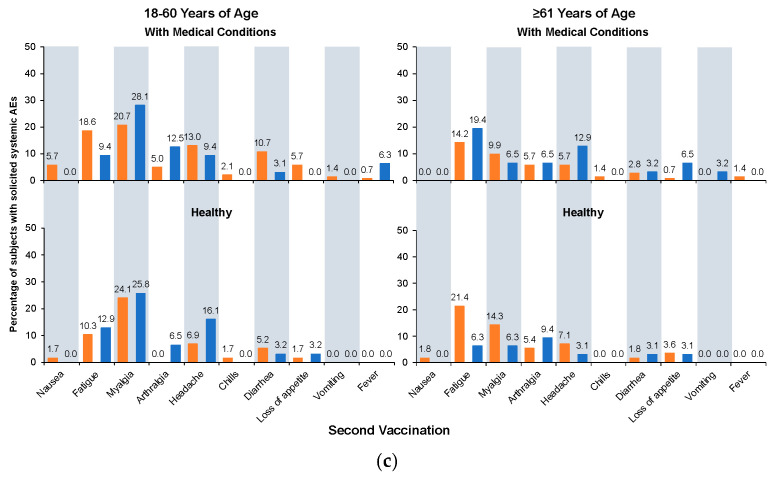
Solicited adverse events (AEs). aH5N1 = adjuvanted H5N1 vaccine; aTIV = adjuvanted trivalent seasonal influenza vaccine. (**a**) Solicited local AEs occurring within 7 days of the first or second vaccination. (**b**) Solicited systemic AEs occurring within 7 days of the first vaccination. (**c**) Solicited systemic AEs occurring within 7 days of the second vaccination. Fever is defined as body temperature ≥ 38 °C.

The frequency of solicited AEs was similar between vaccine groups (Figure 4). Most local AEs were mild or moderate in severity. Pain was the most common local AE and the only one for which severe events were reported. Severe pain was reported by two aH5N1 and one aTIV recipient after the first vaccination and by one aH5N1 and two aTIV recipients after the second vaccination, all of whom were in the 18–60-year age stratum. Fewer solicited local AEs were reported after the second vaccination compared with the first (Figure 4a).

Solicited systemic AEs were more common among younger vs. older subjects in both vaccine groups (Figure 4b,c). Fatigue and myalgia occurred most frequently. The most common severe systemic AEs after the first vaccination were headache, which affected four aH5N1 recipients who were 18–60 years of age with medical conditions but no subjects from other subgroups, and myalgia, which affected one healthy aTIV recipient who was 18–60 years of age but no other subjects from other subgroups. Other severe AEs occurred in <2% of any subgroup. In general, fewer systemic AEs were reported after the second than after the first vaccination (Figure 4c), and rates of severe AEs were also lower after the second vaccination. The majority of solicited systemic events were mild or moderate in nature

In the 18–60 years of age stratum, unsolicited AEs considered at least possibly related to study vaccine occurring between Days 1 and 43 were reported in 6.9% of aH5N1 and 12.9% of aTIV recipients who were healthy and 9.1% and 11.8% of subjects with medical conditions from these respective vaccine groups. Overall, headache was the most common unsolicited AE among healthy subjects in both vaccine groups, whereas diarrhea was most commonly reported among those with medical conditions (Table 4). Among subjects ≥61 years of age, at least possibly related unsolicited AEs were reported by 5.2% and 9.4% of healthy subjects and by 8.1% and 9.7% of subjects with medical conditions from the aH5N1 and aTIV groups, respectively. Nasopharyngitis was the most common unsolicited AE among healthy older aH5N1 recipients, whereas fatigue was most common among those with medical conditions in this group. In the aTIV group, fatigue, arthralgia, and nasopharyngitis were most often reported among healthy older subjects, whereas myalgia and pyrexia were the most frequent unsolicited AEs among those with medical conditions (Table 4).

## 4. Discussion

In this study, two doses of the pre-pandemic aH5N1 vaccine increased antibody titers in younger and older age strata and in both healthy subjects and those with underlying medical conditions. aH5N1 was well tolerated, with a lower frequency of solicited and unsolicited AEs than the adjuvanted seasonal influenza vaccine comparator, aTIV, which may be attributed to lower antigen content. In general, the rate of AEs was lower after the second than after the first aH5N1 vaccination, which may be attributed to the reduced antigen content, and older subjects tended to report fewer AEs than younger subjects. Within each age stratum, the frequencies of AEs among healthy vs. subjects with medical conditions were similar.

This study was conducted as a post-authorization commitment for aH5N1 requested by the CHMP to collect clinical data in adult (18–60 years of age) and older (≥61 years of age) individuals with medical conditions that would make them more likely to suffer from influenza complications. We also collected data from healthy younger and older adult populations as positive controls.

The primary immunogenicity objective of this study was to evaluate immune responses to the homologous Turkey 2005 strain with the HI assay 3 weeks after the second vaccination (Day 43) according to former CHMP criteria for each age group. The HI assay did not perform as seen in other aH5N1 trials [18,19]. In a study published in 2012, 3 weeks after the second dose of aH5N1, HI seroconversion (a ≥four-fold increase in HI titers) was achieved by 56% (95% CI, 49–63%) of adults aged 18–60 years, with a GMR of 7.1 (5.52–9.14), and in adults aged ≥61 years, the seroconversion rate was 50% (95% CI, 43–57%) and GMR was 5.15 (4.15–6.40). CHMP criteria for HI GMR and seroconversion were met in both age groups [18]. In a 2019 study, 85% (97.5% CI, 81–88%) of adults aged 18–64 years and 74% (97.5% CI, 70–77%) of those aged ≥65 years who were vaccinated with aH5N1 achieved HI titers ≥1:40, meeting CHMP criteria [19]. In the current trial, the former CHMP HI criterion for GMR was met for three of the four groups given aH5N1 (healthy subjects in both age strata and subjects with medical conditions who were ≥61 years of age).

The SRH and MN assays used in this study were both validated as sensitive and accurate, and the findings were consistent with previous studies [18,19]. On Day 43, across both age strata, healthy recipients of aH5N1 met all 3 former CHMP SRH criteria (GMR, seroconversion rate, and percentage of subjects with an SRH area ≥ 25 mm^2^), whereas those with comorbid conditions met former CHMP SRH criteria for seroconversion and GMR. Subjects 18–60 years of age with comorbidities in the aH5N1 group and all aH5N1 recipients ≥61 years of age did not meet CHMP criteria for seroprotection (SRH area ≥ 25 mm^2^). There are no CHMP criteria for the MN assay, but the antibody titers measured with this assay showed a robust immune response on Day 43, consistent with the SRH results. As anticipated, low antibody responses were observed following a single vaccination on Day 22. Although the persistence of antibody titers was seen on Day 202 and remained above baseline, a waning response in comparison to Day 43 was observed.

The heterologous findings were encouraging. All aH5N1 recipients met former CHMP SRH seroconversion criteria against the heterologous Vietnam strain, and healthy subjects 18–60 years of age and those ≥61 years of age with medical conditions met the SRH seroconversion criteria against the Anhui strain.

## 5. Conclusions

Across age strata divided between younger (18–60 years of age) and older adults (≥61 years of age), aH5N1 increased antibody responses in both healthy subjects as well as individuals with comorbidities that put them at higher risk of influenza complications. Results obtained with the MN and SRH assays were consistent with previous studies of aH5N1. In line with data obtained from previous aH5N1 studies, the aH5N1 vaccine was shown to have a clinically acceptable safety and tolerability profile.

## Figures and Tables

**Table 1:** Demographic and clinical characteristics at baseline in all enrolled subjects.

	18–60 Years of Age				≥61 Years of Age			
	Medical Conditions		Healthy		Medical Conditions		Healthy	
Characteristic	aH5N1 (n = 146)	aTIV (n = 34)	aH5N1 (n = 59)	aTIV (n = 31)	aH5N1 (n = 149)	aTIV (n = 31)	aH5N1 (n = 58)	aTIV (n = 32)
Mean age ± SD, years	49.8 ± 9.37	46.9 ± 11.59	37.6 ± 11.81	41.1 ± 11.59	68.1 ± 5.53	69.9 ± 6.08	68.9 ± 5.28	69.3 ± 5.63
Mean BMI ± SD, kg/m^2^	29.2 ± 6.82	28.5 ± 6.02	25.1 ± 3.94	26.4 ± 4.32	29.3 ± 5.59	28.7 ± 2.97	27.4 ± 3.58	26.8 ± 4.43
Female, n (%)	51 (34.9)	11 (32.4)	34 (57.6)	19 (61.3)	33 (22.1)	7 (22.6)	35 (60.3)	20 (62.5)
Race and ethnicity								
Black	0	0	1 (1.7)	0	0	0	0	0
White	146 (100)	34 (100)	58 (98.3)	31 (100)	149 (100)	31 (100)	58 (100)	32 (100)
Hispanic or Latino	1 (0.7)	1 (2.9)	0	0	1 (0.7)	0	1 (1.7)	
CCI score, n (%) *								
1	15 (10.3)	9 (26.5)	0	0	0	0	0	0
2	29 (19.9)	5 (14.7)	0	0	0	0	0	0
3	64 (43.8)	13 (38.2)	0	0	0	0	0	0
4	24 (16.4)	4 (11.8)	0	0	51 (34.2)	6 (19.4)	0	0
5	11 (7.5)	3 (8.8)	0	0	55 (36.9)	18 (58.1)	0	0
6	3 (2.1)	0	0	0	39 (26.2)	7 (22.6)	0	0
≥6	0	0	0	0	4 (2.7)	0	0	0
Underlying medical condition, n (%) *								
Chronic pulmonarydisease	31 (21.2)	6 (17.7)	0	0	35 (23.5)	10 (32.3)	0	0
Cardiovascular disease	26 (17.8)	4 (11.8)	0	0	40 (26.8)	6 (19.4)	0	0
Peripheral vasculardisease	5 (3.4)	1 (2.9)	0	0	9 (6.0)	0	0	0
Diabetes	79 (54.1)	22 (64.7)	0	0	57 (38.3)	13 (41.9)	0	0
Renal impairment	5 (3.4)	1 (2.9)	0	0	8 (5.4)	2 (6.5)	0	0

* Not applicable to healthy subjects. aH5N1 = adjuvanted H5N1 pandemic influenza vaccine; aTIV = adjuvanted trivalent seasonal influenza vaccine; BMI = body mass index; CCI = Charlson comorbidity index; SD = standard deviation.

**Table 2:** Antibody responses to heterologous strains.

	18–60 Years of Age		≥61 Years of Age	
Result (95% CI)	Medical Conditions (n = 136)	Healthy (n = 40)	Medical Conditions (n = 140)	Healthy (n = 42)
**Vietnam/2004**				
**MN Assay**				
GMT, Day 1	5.55 (5.22–5.90)	5.27 (4.86–5.71)	6.74 (6.21–7.31)	6.10 (5.33–6.97)
GMT, Day 43	9.42 (8.26–10.76) (n = 133)	12.63 (9.93–16.07) (n = 39)	10.00 (9.02–11.08) (n = 137)	11.36 (8.97–14.39) (n = 41)
GMR, Day 43/Day 1	1.64 (1.43–1.87)	2.30 (1.81–2.93)	1.51 (1.37–1.68)	1.87 (1.48–2.37)
Percentage with MN titers ≥40, Day 43	9 (4.7–15.2)	8 (1.6–20.9)	5 (2.1–10.2)	7 (1.5–19.9)
Percentage with ≥4-fold increase in MN titers, Day 43	7.52 (3.7–13.4)	7.69 (1.6–20.9)	3.65 (1.2–8.3)	4.88 (0.6–16.5)
**SRH Assay**				
GMT, Day 1	7.30 (6.52–8.16)	7.68 (6.31–9.35)	8.01 (7.07–9.07)	8.45 (6.83–10.45)
GMT, Day 43	14.41 (12.51–16.60) (n = 133)	15.63 (11.89–20.53) (n = 39)	12.91 (11.33–14.72) (n = 137)	15.55 (11.72–20.63)
GMR, Day 43/Day 1	2.02 (1.75–2.33)	1.98 (1.50–2.60)	1.58 (1.39–1.80)	1.98 (1.49–2.63)
Percentage with SC, Day 43	51.88 (43.1–60.6)	58.97 (42.1–74.4)	34.31 (26.4–42.9)	48.78 (32.9–64.9)
Percentage with SRH area >25 mm^2^, Day 43	31.58 (23.8–40.2)	30.77 (17–47.6)	24.82 (17.8–32.9)	29.27 (16.1–45.5)
**Anhui/2005**				
**MN Assay**				
GMT, Day 1	5.36 (5.11–5.62)	5.00 (5.00–5.00)	5.53 (5.29–5.79)	5.39 (5.00–5.80)
GMT, Day 43	7.69 (7.03–8.40) (n = 133)	8.15 (6.74–9.85) (n = 39)	8.61 (7.79–9.51) (n = 137)	7.76 (6.42–9.39) (n = 41)
GMR, Day 43/Day 1	1.43 (1.30–1.56)	1.63 (1.35–1.97)	1.57 (1.42–1.73)	1.48 (1.22–1.78)
Percentage with MN titers ≥40, Day 43	3 (0.8–7.5)	3 (0.06–13.5)	4 (1.6–9.3)	2 (0.06–12.9)
Percentage with ≥4-fold increase in MN titers, Day 43	2.26 (0.47–6.5)	2.56 (0.06–13.5)	3.65 (1.2–8.3)	2.44 (0.06–12.9)
**SRH Assay**				
GMT, Day 1	8.19 (7.18–9.34)	5.50 (4.85–6.24)	7.19 (6.47–8.00)	6.66 (5.50–8.06)
GMT, Day 43	12.88 (11.25–14.74) (n = 133)	11.34 (8.97–14.35) (n = 39)	10.30 (9.05–11.74) (n = 137)	8.65 (6.87–10.89) (n = 41)
GMR, Day 43/Day 1	1.61 (1.40–1.84)	1.97 (1.55–2.49)	1.44 (1.26–1.64)	1.31 (1.04–1.64)
Percentage with SC, Day 43	39.85 (31.5–48.7)	48.72 (32.4–65.2)	32.85 (25.1–41.4)	24.39 (12.4–40.3)
Percentage with SRH area >25 mm^2^, Day 43	27.82 (20.4–36.3)	20.51 (9.3–36.5)	18.98 (12.8–26.6)	14.63 (5.6–29.2)

**Table 3:** Overall summary of solicited and unsolicited AEs.

AE, n (%)	aH5N1 (n = 411)	aTIV (n = 128)
Any ^a^	306 (74.5)	109 (85.2)
Solicited AE		
Any	309 (75.2)	109 (85.2)
Local	261 (63.5)	98 (76.6)
Systemic	217 (52.8)	75 (58.6)
Analgesic/antipyretic use	30 (7.3%)	8 (6.3%)
Unsolicited AE		
Any	164 (40.2)	39 (30.5)
Severe	38 (9.3)	8 (6.3)
Related	35 (8.6)	16 (12.5)
Leading to study withdrawal, excluding deaths	7 (1.7)	3 (2.3)
SAEs		
Any	45 (11.0)	3 (2.3)
Related	0	0
Medically attended AE	135 (32.8)	32 (25.0)
AESI	0	0
NOCD	7 (1.7)	3 (2.3)
Death	4 (1.0)	0

AE, adverse event; AESI, adverse event of special interest; NOCD, new onset chronic disease; SAE, serious adverse event. ^a^ Reported by >5% of subjects, excluding SAEs.

**Table 4:** Summary of unsolicited AEs occurring between Days 1 and 43 with frequency ≥5% after any vaccination by MedDRA preferred term.

	All AEs, n (%)				At Least Possibly Related AEs, n (%)			
	Medical Conditions		Healthy		Medical Conditions		Healthy	
	aH5N1	aTIV	aH5N1	aTIV	aH5N1	aTIV	aH5N1	aTIV
**18–60 years of age**	(**n = 143**)	(**n = 34**)	(**n = 58**)	(**n = 31**)	(**n = 143**)	(**n = 34**)	(**n = 58**)	(**n = 31**)
Any	43 (30.1)	8 (23.5)	13 (22.4)	9 (29.0)	13 (9.1)	4 (11.8)	4 (6.9)	4 (12.9)
Diarrhea	5 (3.5)	1 (2.9)	0	0	0	0	0	0
Nasopharyngitis	3 (2.1)	1 (2.9)	0	0	0	0	0	0
Fatigue	3 (2.1)	0	0	1 (3.2)	0	0	0	1 (3.2)
Headache	3 (2.1)	0	3 (5.2)	2 (6.5)	0	0	1 (1.7)	1 (3.2)
Toothache	0	0	2 (3.4)	1 (3.2)	0	0	0	0
Arthralgia	3 (2.1)	0	0	2 (6.5)	0	0	0	0
Food poisoning	0	0	2 (3.4)	0	0	0	0	0
Myalgia	0	0	0	2 (6.5)	0	0	0	1 (3.2)
**≥61 years of age**	(**n = 149**)	(**n = 31**)	(**n = 58**)	(**n = 32**)	(**n = 149**)	(**n = 31**)	(**n = 58**)	(**n = 32**)
Any	45 (30.2)	9 (29.0)	16 (27.6)	3 (9.4)	12 (8.1)	3 (9.7)	3 (5.2)	3 (9.4)
Fatigue	5 (3.4)	1 (3.2)	0	1 (3.1)	4 (2.7)	0	0	1 (3.1)
Arthralgia	3 (2.0)	1 (3.2)	1 (1.7)	1 (3.1)	2 (1.3)	1 (3.2)	0	1 (3.1)
Headache	3 (2.0)	1 (3.2)	0	0	0	0	0	0
Pain in extremity	3 (2.0)	0	0	0	0	0	0	0
Myalgia	2 (1.3)	2 (6.5)	0	0	2 (1.3)	2 (6.5)	0	0
Diarrhea	2 (1.3)	1 (3.2)	0	0	0	0	0	0
Nasopharyngitis	0	0	3 (5.2)	1 (3.1)	0	0	1 (1.7)	1 (3.1)
Hypertension	0	0	2 (3.4)	0	0	0	0	0
Decreased appetite	2 (1.3)	1 (3.2)	0	0	0	0	0	0
Pyrexia	1 (0.7)	2 (6.5)	0	0	0	0	0	0

AE = adverse event; MedRA = Medical Dictionary for Regulatory Activities.

## Data Availability

Data available in EU trials register: EudraCT Number 2011-003603-37—Clinical trial results—EU Clinical Trials Register, and on ClinicalTrials.gov (NCT02091908): Study Details | Safety and Immunogenicity of Two Doses of aH5N1 Vaccine in Adult and Elderly Subjects with and without Underlying Medical Conditions|ClinicalTrials.gov.

## Supplementary Materials

The following supporting information can be downloaded at: https://www.mdpi.com/article/10.3390/vaccines12050481/s1, List of Inclusion and Exclusion Criteria; Table S1: HI Antibody Response Against Homologous Strain (A/turkey/Turkey/1/2005) in the Full Analysis Set; Table S2: Subjects with MN Titers ≥10, ≥20, and ≥80 Against the Homologous Strain (A/turkey/Turkey/1/2005) in the Full Analysis Set.
