# Supplementary material for: Safety, Tolerability, and Immunogenicity of aH5N1 Vaccine in Adults with and without Underlying Medical Conditions
Source: Vaccines (Basel). 2024 Apr 30;12(5):481. doi: 10.3390/vaccines12050481 (PMC11125740; doi:10.3390/vaccines12050481)
Supplement: Supplementary file 1 [file vaccines-12-00481-s001.zip › vaccines-2931751-supplementary.pdf]

Supplement to

Safety, Tolerability, and Immunogenicity of aH5N1 in Adults With and Without Underlying Medical Conditions

INCLUSION AND EXCLUSION CRITERIA

Inclusion Criteria

In order to be eligible for participating in this study, a subject had to meet ALL of the following inclusion criteria in the "All Subjects" section and ALL of the inclusion criteria in the respective section for his/her health status (healthy or with underlying medical condition).

All Subjects

1. Male and female subjects 18 years of age and older at the time of enrollment who were mentally competent, willing and able to understand the nature and risks of the proposed study, and able to sign the consent form prior to study entry.
2. Subjects with a projected life expectancy of 12 months or longer.
3. Subjects who were able to comply with all study procedures and requirements.

Subjects With Underlying Medical Conditions Only

4. Subjects with at least one of the following medical conditions:
   a. Documented underlying chronic respiratory medical condition: chronic pulmonary disease. Classification of severity of severity of chronic obstructive pulmonary disorder (COPD) was based on the 2013 Global Initiative for Chronic Obstructive Lung Disease (GOLD) criteria, including evaluation of force expiratory volume (FEV1) within 3 months prior to enrollment. Eligible subjects had to be classified with either GOLD 2 (moderate) or GOLD 3 (severe) impairment for enrollment (GOLD, 2013).
   b. Underlying cardiovascular medical condition:
      - Documented myocardial infarction (confirmed by at least 2 of the following: symptoms, electrocardiogram (ECG) changes, biochemical markers, echocardiogram findings),
      - Documented congestive heart failure with New York Heart Association (NYHA) functional classification Class II or III,
   c. Documented peripheral vascular disease including a Rutherford symptom score of 2 (moderate claudication) or higher,
   d. Documented diabetes mellitus with hemoglobin A1c ⩾7 to <10% within 3 months prior to enrollment,
   e. Documented moderate to severe renal impairment as reflected by glomerular filtration rate (GFR) of <60 mL/min/1.73 m2 within 3 months prior to enrollment
      OR
      Currently receiving hemodialysis treatments.
5. Subjects with a CCI score of 6 or below.

Healthy Subjects Only

6. Subjects who were in good health as determined by the outcome of medical history, physical assessment, and clinical judgment of the Investigator.

Exclusion Criteria

In order to be eligible for participating in this study, a subject should have met NONE of the following exclusion criteria in the "All Subjects" section and NONE of the exclusion criteria in the respective section for his/her health status (healthy or with underlying medical condition):

All Subjects

1. Subjects who were not able to follow all the required study procedures for the whole period of the study.
2. Subjects with behavioral or cognitive impairment or psychiatric disease that, in the opinion of the Investigator, could interfere with the subject's ability to participate in the study.
3. Subjects who were hospitalized or residing in a nursing care facility.
4. Subjects who were planning to change their home address due to relocation during the course of the study and could be unavailable for follow-up.
5. Subjects with any fatal prognosis of an underlying medical condition (<12 months life expectancy).
6. Subjects with any progressive or severe neurologic disorder, seizure disorder, or history of Guillain-Barré syndrome.
7. Subjects with known human immunodeficiency virus (HIV) infection or HIV-related disease.
8. Subjects who received other (nonstudy) vaccines within 7 days of either Day 1 or Day 22 vaccination.
9. Subjects who had ever received an H5N1 vaccine.
10. Subjects who received another investigational product within 30 days prior to Day 1 or before completion of the safety follow-up period in another study and who were unwilling to refuse participation in another clinical study at any time during the conduct of this study.

    Note: Concomitant participation in an observational study (not involving drugs, vaccines, or medical devices) was acceptable.
11. Subjects with a history of any anaphylaxis, serious vaccine reactions, or hypersensitivity to any of the following: influenza viral proteins, excipient(s) of the study or reference vaccine, eggs (including ovalbumin), or chicken protein.
12. Subjects with a history of (or current) drug or alcohol abuse that, in the Investigator's opinion, could have interfered with the subject's safety or evaluation of the study objectives.
13. Subjects who had to undergo surgery planned during the study period that, in the Investigator's opinion, could have interfered with the study visit schedule.
14. Subjects who were a member of the research staff or had relatives who were members of the research staff (research staff being defined as individuals with direct contact with study subjects or study site personnel with access to any study document containing subject information, including receptionists, persons scheduling appointments or making screening calls, regulatory specialists, or laboratory technicians). Hospital personnel, health care professionals, and their relatives who were not involved in this clinical study were allowed for inclusion.
15. Female subjects of childbearing potential (status post onset of menarche and not meeting any of the following conditions: menopausal for at least 2 years, status post bilateral tubal ligation for at least 1 year, status post bilateral oophorectomy, or status post hysterectomy) who were sexually active and had not used for at least 2 months prior to study entry one or more of the following acceptable contraceptive methods:
    a. Hormonal contraceptive (oral, injection, transdermal patch, implant, cervical ring),
    b. Barrier (condom with or without spermicide or diaphragm with spermicide) each and every time during intercourse,
    c. Intrauterine device,
    d. Monogamous relationship with vasectomized partner (partner must have been vasectomized for at least 6 months prior to the subject's study entry),

    e. Abstinence, if not sexually active, at least 2 months before study entry and at least 2 months after study entry (through Day 60 of study participation).

16. Female subjects of childbearing potential (as defined above) who had a positive pregnancy test prior to study entry, who were nursing (breastfeeding), or who were sexually active and had not used or did not plan to use acceptable contraceptive measures through Day 60 of study participation.
17. Subjects with a body temperature ≥38°C (≥100.4°F) (as measured orally) within 3 days of intended study vaccination.
18. Subjects with a medical history or any illness that, in the opinion of the Investigator, posed an additional risk to the subjects due to participation in the study.

Subjects With Underlying Medical Conditions Only

19. Subjects with a CCI score greater than 6.

Healthy Subjects Only

20. Subjects who received or were planning to receive blood, blood products, and/or plasma derivatives or any parenteral immunoglobulin preparation within 12 weeks prior to Day 1 or during the full length of the study.
21. Subjects with a known bleeding diathesis or any condition that could be associated with a prolonged bleeding time.
22. Subjects who had a malignancy (excluding nonmelanotic skin cancer) or lymphoproliferative disorder within the past 5 years;
23. Subjects who were receiving cancer chemotherapy, oral or systemic corticosteroids (topical, inhaled, and intranasal corticosteroids are permitted), or other immunosuppressive agents.
24. Subjects with any symptoms or diagnosis of any of the underlying conditions as described in Inclusion Criterion 4.

General note: There could have been instances when subjects met all entry criteria except one that relates to transient clinical circumstances (eg, body temperature elevation or recent use of excluded medication or vaccine). Under these circumstances, a subject could be considered eligible for study enrollment if the appropriate window for delay had passed, inclusion/exclusion criteria were rechecked, and the subject was confirmed to be eligible.

Table S1. HI Antibody Response Against Homologous Strain (A/turkey/Turkey/1/2005) in the Full Analysis Set

	**18-60 years of age**				**≥61 years of age**			
	**With medical conditions**		**Healthy**		**With medical conditions**		**Healthy**	
**Result (95% CI)**	**aH5N1 (n=136)**	**aTIV (n=33)**	**aH5N1 (n=40)**	**aTIV (n=21)**	**aH5N1 (n=140)**	**aTIV (n=29)**	**aH5N1 (n=42)**	**aTIV (n=27)**
**GMT**								
Day 1	5.47 (5.12-5.84)	5.27 (4.61-6.02)	5.00 (5.00-5.00)	5.00 (5.00-5.00)	5.80 (5.36-6.28)	6.20 (5.21-7.38)	5.21 (4.86-5.58)	5.13 (4.71-5.59)
Day 22	8.43 (7.22-9.85) (n=130)	5.75 (4.20-7.87) (n=32)	9.73 (7.19-13.16) (n=38)	5.90 (3.93-8.86)	9.31 (8.01-10.83) (n=136)	7.23 (5.21-10.03)	8.61 (6.63-11.19) (n=41)	5.81 (4.21-8.03)
Day 43	13.17 (11.08-15.65) (n=133)	6.13 (4.33-8.67)	17.20 (12.36-23.92) (n=39)	5.43 (3.46-8.51)	15.42 (12.78-18.61) (n=137)	6.96 (4.56-10.64) (n=27)	12.66 (9.47-16.91) (n=41)	6.73 (4.71-9.62) (n=27)
Day 202	6.68 (6.15-7.26) (n=128)	5.62 (4.76-6.64) (n=32)	6.62 (5.64-7.77) (n=37)	5.00 (4.04-6.18) (n=21)	8.41 (7.45-9.49) (n=131)	6.85 (5.28-8.90) (n=28)	6.85 (5.92-7.92)	5.19 (4.33-6.23)
**GMR**								
Day 22/ Day 1	1.57 (1.34-1.83) (n=130)	1.07 (0.78-1.46) (n=32)	1.95 (1.44-2.63) (n=38)	1.18 (0.79-1.77)	1.59 (1.37-1.85) (n=136)	1.24 (0.89-1.72)	1.66 (1.28-2.16) (n=41)	1.12 (0.81-1.55)
Day 43/ Day 1	2.42 (2.04-2.88) (n=133)	1.13 (0.80-1.60)	**3.44** (2.47-4.78) (n=39)	1.09 (0.69-1.70)	**2.64** (2.19-3.18) (n=137)	1.19 (0.78-1.82) (n=27)	**2.44** (1.83-3.26) (n=41)	1.30 (0.91-1.86)
Day 202/ Day 1	1.23 (1.13-1.33)	1.03 (0.87-1.22)	1.32 (1.13-1.55)	1.00 (0.81-1.24)	1.43 (1.27-1.61)	1.17 (0.90-1.51)	1.32 (1.14-1.53)	1.00 (0.84-1.20)
**Percentage with SC**								
Day 22	13.85 (8.4-21) (n=130)	3.13 (0.08-16.2) (n=32)	21.05 (9.6-37.3) (n=38)	4.76 (0.12-23.8)	13.24 (8-20.1) (n=136)	3.45 (0.09-17.8)	9.76 (2.7-23.1) (n=41)	3.70 (0.09-19)
Day 43	26.47 (19.3-34.7)	3.03 (0.08-15.8)	37.50 (22.7-54.2)	0	27.86 (20.6-36.1)	3.45 (0.09-17.8)	21.43 (10.3-36.8)	3.70 (0.09-19)
Day 202	3.13 (0.9-7.8)	0	0	0	8.40 (4.3-14.5)	0	0	0
**Percentage with HI titer ≥1:40**								
Day 1	2 (0.46-6.3)	0	0	0	2 (0.44-6.1)	0	0	0
Day 22	15 (9.7-22.8) (n=130)	3 (0.08-16.2) (n=32)	21 (9.6-37.3) (n=38)	5 (0.12-23.8)	15 (9.8-22.6) (n=136)	7 (0.8-22.8)	12 (4.1-26.2) (n=41)	4 (0.09-19)
Day 43	30 (22.4-38.6) (n=133)	6 (0.7-20.2)	38 (23.4-55.4) (n=39)	0	33 (25.1-41.4) (n=137)	7 (0.9-24.3) (n=27)	24 (12.4-40.3) (n=41)	4 (0.09-19)
Day 202	5 (2.2-10.9)	0	0	0	13 (7.7-20)	0	2 (0.06-12.6)	0

Boldface indicates that former CHMP criteria were met.

aH5N1 = adjuvanted H5N1 vaccine; aTIV = adjuvanted trivalent seasonal influenza vaccine; CHMP = Committee for Medicinal Products for Human Use; CI = confidence interval; GMR = geometric mean ratio; GMT = geometric mean titer; HI hemagglutination inhibition; SC = seroconversion.

Table S2. Subjects with MN Titers ≥10, ≥20, and ≥80 Against the Homologous Strain (A/turkey/Turkey/1/2005) in the Full Analysis Set

	**18-60 years of age**				**≥61 years of age**			
	**With medical conditions**		**Healthy**		**With medical conditions**		**Healthy**	
**Result (95% CI)**	**aH5N1 (n=136)**	**aTIV (n=33)**	**aH5N1 (n=40)**	**aTIV (n=21)**	**aH5N1 (n=140)**	**aTIV (n=29)**	**aH5N1 (n=42)**	**aTIV (n=27)**
**Number (%) with MN titers ≥10 [95% CI]**								
Day 1	95 (70) [61.4-77.4]	26 (79) [61.1-91]	9 (23) [10.8-38.5]	6 (29) [11.3-52.2]	108 (77) [69.3-83.8]	28 (97) [82.2-99.9]	17 (40) [25.6-56.7]	15 (56) [35.3-74.5]
Day 22	119 (92) [85.4-95.7] (n=130)	30 (94) [79.2-99.2] (n=32)	24 (63) [46-78.2] (n=38)	9 (43) [21.8-66]	124 (91) [85.1-95.4] (n=136)	29 (100) [88.1-100]	23 (56) [39.7-71.5] (n=41)	15 (56) [35.3-74.5]
Day 43	127 (95) [90.4-98.3] (n=133)	31 (94) [79.8-99.3]	36 (92) [79.1-98.4] (n=39)	10 (48) 25.7-70.2	130 (95) [89.8-97.9] (n=137)	27 (100) [87.2-100] (n=27)	31 (76) [59.7-87.6] (n=41)	14 (52) [31.9-71.3]
Day 202	124 (97) [92.2-99.1] (n=128)	27 (84) [67.2-94.7] (n=32)	29 (78) [61.8-90.2] (n=37)	9 (43) [21.8-66]	122 (93) [87.4-96.8] (n=131)	24 (86) [67.3-96] (n=28)	28 (67) [50.5-80.4]	13 (48) [28.7-68.1]
**Number (%) with MN titers ≥20 [95% CI]**								
Day 1	35 (26) [18.6-33.9]	12 (36) [20.4-54.9]	0	2 (10) [1.2-30.4]	53 (38) [29.8-46.4]	13 (45) [26.4-64.3]	12 (29) [15.7-44.6]	6 (22) [8.6-42.3]
Day 22	92 (71) [62.2-78.4] (n=130)	26 (81) [63.6-92.8] (n=32)	14 (37) [21.8-54] (n=38)	5 (24) [8.2-47.2]	91 (67) [58.3-74.7] (n=136)	18 (62) [42.3-79.3]	17 (41) [26.3-57.9] (n=41)	7 (26) [11.1-46.3]
Day 43	113 (85) [77.7-90.6] (n=133)	23 (70) [51.3-84.4]	36 (92) [79.1-98.4] (n=39)	6 (29) [11.3-52.2]	116 (85) [77.5-90.3] (n=137)	17 (63) [42.4-80.6] (n=27)	23 (56) [39.7-71.5] (n=41)	7 (26) [11.1-46.3]
Day 202	86 (67) [58.3-75.2] (n=128)	14 (44) [26.4-62.3] (n=32)	18 (49) [31.9-65.6] (n=37)	1 (5) [0.12-23.8]	98 (75) [66.5-82] (n=131)	14 (50) [30.6-69.4] (n=28)	17 (40) [25.6-56.7]	6 (22) [8.6-42.3]
**Number (%) with MN titers ≥80 [95% CI]**								
Day 1	7 (5) [2.1-10.3]	2 (6) [0.7-20.2]	0	0	8 (6) [2.5-10.9]	0	3 (7) [1.5-19.5]	1 (4) [0.09-19]
Day 22	26 (20) [13.5-27.9] (n=130)	7 (22) [9.3-40] (n=32)	7 (18) [7.7-34.3] (n=38)	2 (10) [1.2-30.4]	15 (11) [6.3-17.5] (n=136)	3 (10) [2.2-27.4]	9 (22) [10.6-37.6] (n=41)	1 (4) [0.09-19]
Day 43	56 (42) [33.6-51] (n=133)	6 (18) [7-35.5]	13 (33) [19.1-50.2] (n=39)	0	52 (38) [29.8-46.6] (n=137)	1 (4) [0.09-19] (n=27)	14 (34) [20.1-50.6] (n=41)	1 (4) [0.09-19]
Day 202	17 (13) [7.9-20.4] (n=128)	1 (3) [0.08-16.2] (n=32)	5 (14) [4.5-28.8] (n=37)	0	14 (11) [6-17.3] (n=131)	0 (n=28)	7 (17) [7-31.4]	1 (4) [0.09-19]

aH5N1 = adjuvanted H5N1 vaccine; aTIV = adjuvanted trivalent seasonal influenza vaccine; CI = confidence interval; FAS = full analysis set; MN = microneutralization.
